# Myxomatous Mitral Valve Disease with Mitral Valve Prolapse and Mitral Annular Disjunction: Clinical and Functional Significance of the Coincidence

**DOI:** 10.3390/jcdd8020009

**Published:** 2021-01-24

**Authors:** Nina C. Wunderlich, Siew Yen Ho, Nir Flint, Robert J. Siegel

**Affiliations:** 1 Cardiovasclar Center Darmstadt, 60431 Darmstadt, Germany; 2 Cardiac Morphology Unit, Royal Brompton Hospital, London SW3 6NP, UK; Yen.ho@imperial.ac.uk; 3 Smidt Heart Institute, Cedars-Sinai Medical Center, Los Angeles, CA 90048, USA; flintn@gmai.com (N.F.); Robert.siegel@cshs.org (R.J.S.)

**Keywords:** mitral valve, primary mitral regurgitation, myxomatous mitral valve disease, mitral valve prolapse, mitral annular disjunction, MitraClip

## Abstract

The morphological changes that occur in myxomatous mitral valve disease (MMVD) involve various components, ultimately leading to the impairment of mitral valve (MV) function. In this context, intrinsic mitral annular abnormalities are increasingly recognized, such as a mitral annular disjunction (MAD), a specific anatomical abnormality whereby there is a distinct separation between the mitral annulus and the left atrial wall and the basal portion of the posterolateral left ventricular myocardium. In recent years, several studies have suggested that MAD contributes to myxomatous degeneration of the mitral leaflets, and there is growing evidence that MAD is associated with ventricular arrhythmias and sudden cardiac death. In this review, the morphological characteristics of MAD and imaging tools for diagnosis will be described, and the clinical and functional aspects of the coincidence of MAD and myxomatous MVP will be discussed.

## 1. Introduction

Since the recognition that mitral valve (MV) leaflets can be affected by myxomatous degeneration (myxomatous mitral valve disease (MMVD)) [1,2], with the main functional feature being mitral valve prolapse (MVP) [3] and consequent mitral regurgitation (MR), our understanding of MMVD and MVP has deepened over the last decades—in particular, due to the finding that the MV annulus is a complex three-dimensional (3D) saddle-shaped structure [4]. In the past, however, the focus of research has been on aspects related to patient outcomes, such as mortality, in relation to the severity of MR [5], the benefit of MV reconstruction versus MV replacement [6], and improved outcome due to early surgery [7].

Yet, MMVD is more complex than isolated MVP, as the morphological changes involve various components of the MV apparatus, ultimately leading to impairment of the MV function. In this context, intrinsic mitral annular abnormalities are increasingly recognized and have clinical ramifications [8,9,10].

In this article, we focus on mitral annular disjunction (MAD), a specific anatomical abnormality associated with MVP characterized by a distinct separation between the mitral annulus and the left atrial wall and the basal portion of the posterolateral left ventricular (LV) myocardium. The first description of MAD was reported in 1986 by Hutchins et al. [11], who found that MAD was present in 92% of 25 autopsy examined hearts with MVP. However, MAD was initially given little attention, since the association between MAD and a worse outcome was not identified. However, more recently, studies indicate that MAD has a greater clinical relevance than initially thought [12,13,14,15]. MAD leads to hypermobility of the MV annulus, contributes to myxomatous degeneration of the mitral leaflets, and there is growing evidence that MAD is associated with ventricular arrhythmias and sudden cardiac death (SCD) [16].

MAD can also occur in normal subjects as a normal anatomical variation of the mitral annulus fibrosus [15], but the combined occurrence of a MVP and MAD is more common. Dejgaard et al. found MVP in 78% (90/116) of patients with MAD [12]. Others reported a prevalence of MAD in 42% [14] to >90% of patients with MMVD and MVP [11,17]. Women seem to be affected more often than men [11,15,16], and it is frequently associated with chest pain [16].

In the following, the morphological characteristics of MAD and imaging tools for diagnosis will be described, and the clinical and functional aspects of the coincidence of MAD and myxomatous MVP will be discussed based on two case studies.

### 1.1. Morphological Characteristics of a MAD

**Figure 1:**
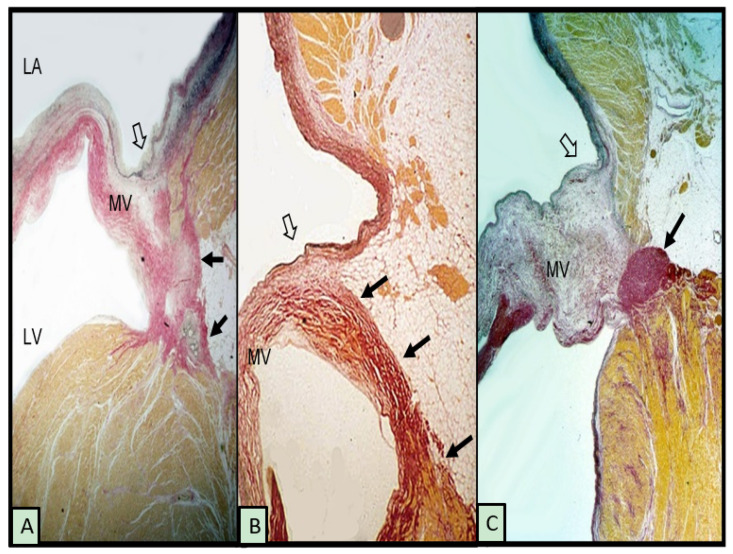
Histological sections from 3 hearts showing the posterior leaflet at the mitral hinge line (open arrows) and variations in mitral annular disjunction (small black arrows). (**A**,**B**) have normal leaflets. The myxomatous leaflet in (**C**) is hinged to the atrial wall and a cord-like annulus (arrow). The elastic van Gieson stain colors fibrous tissue red, myocardium yellow, and elastic dark blue or black. LV = left ventricle, LA = left atrium, and MV = mitral valve.

Although the term annulus implies a complete ring or fibrous cord-like structure around the mitral orifice that hinges the leaflet while separating the adjacent atrial and ventricular walls, this arrangement is uncommon. McAlpine [18] in 1975 asserted that the mitral annulus does not exist as a complete ring, although the term remains in use, as in this article, to refer to the attachment, or hinge line, of the leaflet at the atrioventricular junction. His dissections elegantly revealed a membrane-like structure more ventricular to the hinge line, which he termed the subvalvar segment of the aorto-ventricular membrane around the ostium of the LV. Indeed, subsequent anatomical and imaging studies have shown considerable variability in the attachment of the posterior leaflet at the hinge line [11,19,20]. When viewed on histological sections, instead of a discrete fibrous nodule representing a cord, often, there is a thin, fibrous membrane separating the atrial and ventricular walls. In some hearts, the leaflet is hinged to the atrial wall or the ventricular wall (Figure 1A,B). The gap between is filled with fibro-fatty tissues of the atrioventricular groove. In MAD associated with MMVD, there is a distinct separation conceivably, in part, also due to the thickness of the leaflet (Figure 1C).

### 1.2. Functional Effects of a MAD on the Mitral Annulus

Since the MV annulus has no dynamics of its own, the movement and contraction pattern of the annulus depends on the contractility of the LV myocardium with which the annulus is normally connected, as well as on the movements of the aortic root [21].

Thus, in normal subjects, the MV annulus moves in systole towards the LV apex and in diastole towards the left atrium [22]. In addition, normal MV annular dynamics are characterized by early systolic contraction and deepening of the saddle shape, both factors that contribute significantly to MV competence in the early phase of the LV contraction [8]. Later, in systole, the annulus expands again and reaches almost diastolic dimensions, but since this expansion is limited by the connection to the LV myocardium, there are no functional consequences [8,23]. The dynamics of the mitral annulus are important to ensure a balanced distribution of the mechanical stress exerted by the LV on the MV.

In the presence of MAD, the annulus is functionally decoupled from the LV, and a paradoxical annular dynamic occurs as the annulus moves in concordance with the left atrium during the cardiac cycle and not with the LV. Thus, expanding and flattening of the annulus occurs in systole, causing the section of the LV wall adjacent to the MAD to move outwards in systole and inwards in diastole [14]. Such systolic flattening of the MV annulus increases the mechanical stress on the mitral leaflets [10,24] and chordae tendineae [10], which can accelerate the degenerative process.

Since the region between the aorta and the anterior MV leaflet (aorto-mitral continuity) is fibrous tissue between two robust fibrous trigones, this region is less prone to dilation. MAD is therefore predominantly found in the area of the posterior (mural) leaflet (specifically, the lateral (P1) and middle (P2) posterior scallops are affected [25]), where the annulus is adjacent to the myocardium of the LV [17,26]. Thus, the posterior part of the MV annulus seems to be a weak point for the effects of mechanical stress in the presence of a MAD.

The extent of a MAD is variable and is determined by the distance from the insertion of the posterior leaflet to the LA wall to the transition of the LA wall into the LV wall, which can range from a few millimeters to more than 10 mm [14,26], and by the circumferential extension.

### 1.3. Imaging Modalities to Diagnose MAD

The diagnosis of a MAD is made in systole. It cannot be made in diastole, because the myocardium of the LV is then appropriately positioned under the MV annulus. During systole, the annulus “slides” when the posterolateral portion of the LV contracts and the detachment from the LV myocardium becomes visible. This dynamic nature of a MAD explains why the diagnosis is difficult to make when the heart does not contract—for example, during heart surgery, when the surgeon is viewing the MV from the atrium (in this case, a MAD can only be diagnosed by a superior displacement or atrialization of the base of the posterior leaflet [26,27])—or in pathological studies [11,19].

Noninvasive imaging modalities that allow for an assessment of the MV apparatus and surrounding structures during the cardiac cycle, such as transthoracic (TTE), transesophageal echocardiography (TEE), cardiac computed tomography (CCT), and cardiac magnetic resonance (CMR) imaging, can be used for the detection and quantitative analysis of a MAD.

However, regardless of the imaging modality used, the posterior annulus should be carefully assessed for the presence of a MAD, as it can easily be overlooked. Recognition on its relevancy is not yet widespread.

The diagnosis of a MAD is generally made by measuring the distance between the posterior leaflet insertion into the left atrial wall and the base of the LV free wall in systole. However, the cutoff for diagnosing is not uniformly defined. In the original histological description by Hutchins et al. [11], a MAD was diagnosed if there was a wide separation ≥5 mm. This definition was also adopted in 2D TEE [26,27] and 3D TEE studies [14], whereas others proposed a threshold of ≥2 mm for 2D TTE measurements [15,17]. The prevalence of MAD in MMVD with MVP is therefore described differently, which may be partly due to the nonuniform definition and partly to the use of different imaging modalities (Table 1). In a pooled data analysis incorporating detection rates with different imaging modalities, the rate of a MAD in studies of MMVD patients was 50.8% (66/130; three studies), and among patients with MVP, the rate was 32.6% (95/291; three studies) [28].

### 1.4. Echocardiography

Detecting a MAD by echocardiography requires a careful frame-by-frame analysis of high-resolution images.

**Figure 2:**
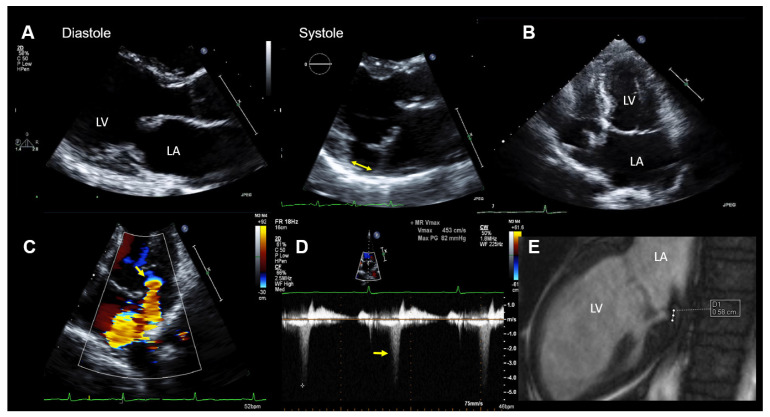
Case 1: Summary of the key findings from multimodality imaging. (**A**) The mitral annular disjunction (MAD) (yellow double arrow) is only visible in systole in a parasternal long-axis transthoracic echocardiography (TTE) view. (**B**) A mildly enlarged left atrium is seen in a 4-chamber TTE view. The MAD is not clearly visualized. (**C**) The proximal isovelocity surface area (PISA) zone (yellow arrow) is seen. A PISA radius of 4 mm and a vena contracta of 3.5 mm indicated nonsevere mitral regurgitation (MR). (**D**) MR occurs only in the late-systole (yellow arrow) in Doppler imaging. (**E**) The MAD is measured in cardiac magnetic resonance imaging (white dots = 5.8 mm). LA = left atrium and LV = left ventricle.

**Figure 3:**
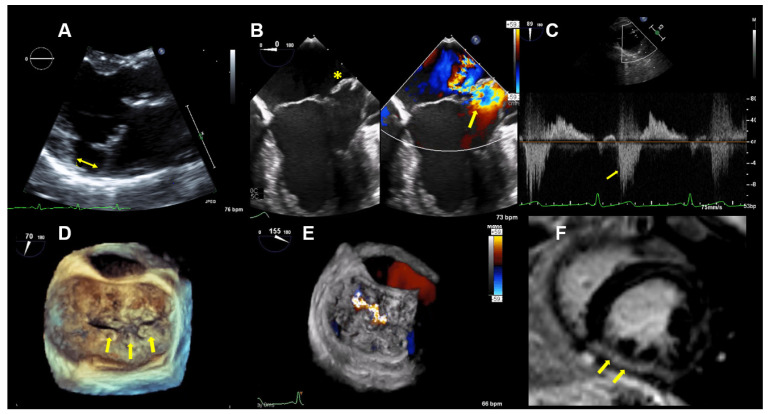
Case 2: Summary of the key findings from multimodality imaging. (**A**) The mitral annular disjunction (yellow double arrow) is visualized in a 3D transthoracic echocardiography view. (**B**) A 4-chamber transesophageal echocardiographic (TEE) view shows the bi-leaflet prolapse, which is more pronounced in the posterior leaflet (yellow asterisk), thus resulting in an eccentric mitral regurgitation (MR) jet, as seen in color Doppler (right; yellow arrow). (**C**) The pulsed-wave Doppler profile derived in the left superior pulmonary vein shows a reverse systolic flow (yellow arrow) as a sign of severe MR. (**D**) Larger prolapse segments are marked with a yellow arrow in a 3D TEE view. (**E**) The MR is seen in a 3D TEE enface view with color Doppler. (**F**) Cardiac magnetic resonance imaging reveals focal mid-wall and subendocardial fibrosis in the mid-inferior wall (yellow arrows).

When 2D TTE imaging is used, a MAD is assessed by measuring the distance from the site of the posterior leaflet insertion into the left atrial wall (upper border of the disjunction) to the connection between the left atrium and the ventricular myocardium (lower border of the disjunction). This should be performed in a parasternal long axis TTE view at end-systole (see, also, Figure 2A and Figure 3A). Carmo et al. found a MAD prevalence of 55% (21/38patients) in patients with MMVD and MVP examined by TTE [16].

Konda et al. studied 185 patients with severe MR. They used a threshold of ≥2 mm to identify a MAD by TTE. In those with fibroelastic deficiency, a MAD was found in 24%, whereas in those with MMVD (Barlow’s disease), 90% had a MAD. The prolapse was localized posteriorly in 77.8% of cases [17].

In the largest 2D TTE study of MAD to date, 979 patients with either MMVD (Barlow’s disease (637 patients) or fibroelastic deficiency (342 patients), a lower than previously reported prevalence of MAD of any length was found (MMVD: 21.8%). The maximum disjunction distance in patients with MMVD was 6.7 ± 2.2 mm, and the main MAD localization was in the region of the lateral (P1) and middle (P2) segments of the posterior leaflet (69.2%) compared to the medial (P3) segment (37.7%) [25].

Using 2D TEE, the degree of annular displacement can be best measured at the P2 level by using a 4-chamber mid-esophageal view at 0 degrees during systole [26,27]. Using a threshold of ≥5 mm, Eriksson et al. found that a MAD was present at the base of the posterior leaflet in 98% of patients with advanced MMVD and in 9% of patients with mild/moderate MMVD. There was a significant correlation between the magnitude of the disjunction and the number of prolapsing segments [26].

Real-time 3D TEE (RT-3D TEE) allows for a more detailed and quantitative analysis of the mitral annular dimensions and dynamics than 2D TEE [8,9,10,31,32].

A RT-3D TEE study comparing the dynamics of the mitral annulus in normal patients and in patients with MMVD showed that, in contrast to normal subjects, the MV annulus in MMVD is still dynamic, but there is no early systolic area contraction and no deepening of the saddle shape, despite a similar magnitude of ventricular contraction, indicating functional ventricular–annular decoupling. In late-systole, the saddle shape deepens, but there is significant enlargement of the annular area, which may also contribute to MV incompetence [8]. By using multiplanar reconstruction, the spatial relation of the posterior leaflet attachment to the left atrial wall and the LV basal myocardium can be examined in multiple reconstructed radial planes rotated around the long axis of the MV. Thus, not only the disjunction distance but, also, the circumferential extent of the MAD can be assessed [14]. Using this imaging approach, 42 of 101 patients with a MVP (42%) had a MAD (using a threshold of ≥5 mm), as reported by Lee et al. [14]. The mean disjunction distance was 8.9 mm (6.3 to 10.7 mm), and the circumferential extension of the MAD was 87° ± 41°. The 3D extent of the MAD correlated significantly with abnormal annular dynamics and a larger regurgitant orifice [14].

### 1.5. Cardiac Computed Tomography (CCT)

CCT can also be used to readily identify a MAD by taking advantage of the 3D nature of this modality. Putnam et al. reported on 90 retrospectively studied patients with MVP who underwent a preoperative CCT. The presence and degree of the MAD was assessed by rotating the view plane around the center of the MV to visualize the disjunction along the annular circumference. A MAD was identified in 20% of the patients, and it was typically located adjacent to a prolapsed or flail posterior mitral leaflet scallop. Of these patients, 75% had a maximum disjunction distance >4.8 mm and 90% >3.8 mm. The presence of a MAD was associated with the female gender, a smaller annulus size, and a greater posterior leaflet length [29].

### 1.6. Cardiac Magnetic Resonance (CMR) Imaging

In a study comparing different imaging modalities (CMR versus. TEE versus TTE) in the detection of a MAD, a significantly higher prevalence of a MAD of any length (cut-off value was ≥2 mm) was found in 131 patients with MVP by CMR imaging (42.0%, 25.5%, and 17.3% by CMR, TEE, and TTE, respectively). The MAD distance was 5.0 (4.0–7.0), 7.0 (5.0–8.0), and 8.0 (7.0–10.0) mm by CMR, TEE, and TTE, respectively. The agreement on MAD identification and on MAD measurement was moderate between TTE and CMR and good between TEE and CMR [30].

A CMR study by Dejgaard et al. demonstrated that the circumferential extension of MAD is variable and varies between 30° and 240° (median 150°), which means that a MAD can take up to two-thirds of the circumference. A MAD was found exclusively along the circumference of the posterior leaflet [12].

CMR imaging has advantages in identifying MAD, as well as providing additional information about the distribution and extent of myocardial fibrosis [33]. Since echocardiography is essential for the morphological and hemodynamic characterizations of MV disease, an integrated multimodality imaging approach seems to be the best to assess patients with MVP and MAD.

## 2. Case Descriptions

### 2.1. Case 1

The patient is a 47-year-old yoga teacher who suffered SCD during one of her yoga classes. Bystander cardiopulmonary resuscitation was performed; she was found to be in ventricular fibrillation and was successfully defibrillated by the paramedics. Prior to this episode, the patient was entirely asymptomatic and had excellent functional capacity.

At the hospital, a physical examination was normal other than mid-systolic click and a mid-late systolic murmur upon cardiac auscultation. The ECG showed no abnormalities, except for frequent polymorphic premature ventricular contractions (PVTs). The laboratory results were within the normal limits. A TTE demonstrated normal LV size and function. The mitral valve appeared myxomatous, and bi-leaflet MVP was present. MR was purely late-systolic (which confirmed the auscultation findings), and by the multimodal quantification of MR, it was moderate. In addition, a MAD was seen. The echocardiographic findings are summarized in Figure 2.

Coronary heart disease was excluded with computed tomography coronary angiography. Due to the aborted SCD and the frequent premature ventricular contractions (PVCs) on the ECG, as well as MAD with MVP, an electrophysiological study was performed, which showed inducible polymorphic ventricular tachycardia (VT).

CMR imaging confirmed the diagnosis of moderate MR (regurgitation fraction was 24%) with mild mitral annular dilation and the presence of a MAD (Figure 2E). The overall LVEF and right ventricular ejection fraction was normal, but a mild hypokinesis of the inferolateral wall was described. In addition, late-gadolinium enhancement CMR imaging showed an increased late-gadolinium uptake in the inferior and inferolateral walls consistent with focal myocardial fibrosis.

After the completion of her diagnostic evaluation, the patient underwent automatic implantable cardioverter defibrillator (AICD) implantation and treatment with a betablocker (Atenolol). During her subsequent five-year follow-up, she has done well, having only occasional palpitations but no dyspnea and no chest pain. In addition, she has not had any further presyncope, syncope, VT detected, or AICD discharges.

### 2.2. Case 2

This patient is a 46-year-old woman with known bi-leaflet prolapse and a known MAD. She has occasional palpitations and mild dyspnea on exertion but was able to function as a yoga and Pilates instructor. She had serial TTE examinations over a period of eight years to monitor her moderate MR. Over this time period, a reduction of the LVEF from 68% to 60% was observed, as well as a slight increase of the LV-end systolic diameter from 37 to 40 mm. Brain natriuretic peptide levels were in the normal range, with 89 pg/mL.

TTE and TEE imaging confirmed the diagnosis of a bi-leaflet prolapse with myxomatous changes of both mitral leaflets (Barlow’s disease) and significant MR (Figure 3). A Zio XT patch monitor (iRhythm Technologies, San Francisco, CA, USA) showed frequent PVCs, nonsustained ventricular tachycardia (nsVT), and supraventricular tachycardia (SVT). Betablocker therapy was initiated.

A stress echocardiography revealed above-average functional capacity. However, she had evidence of an impaired contractile reserve. Namely, she did not have an augmentation of her LVEF with exercise—the increase was <5% during maximal stress. The peak exercise systolic pulmonary artery pressure was 43 mmHg. There were no signs of a stress-related ischemia.

CMR imaging confirmed a mildly impaired LV function (LVEF 53%) and a regurgitant fraction of 45% consistent with moderate-severe MR. The extracellular volume fraction was 34% (normal limit <30%), consistent with diffuse myocardial fibrosis. Focal mid-wall and subendocardial fibrosis was observed in the mid-inferior wall (Figure 3F).

Regarding the decision-making process, different considerations played a role:

(1) Diffuse myocardial fibrosis, as seen in CMR imaging in this patient, is known to be an independent predictor of increased adverse clinical outcomes in patients with chronic MR undergoing MV repair [34,35] and is associated with LV reverse remodeling after MV surgery [35,36].

(2) A lack of contractile reserve is known to be independently associated with a two-fold increase in the risk of cardiac events in patients with primary MR [37] and to be an independent predictor of LV dysfunction (LVEF <50%) after MV surgery in patients with severe primary MR [38].

(3) Even though modern reconstructive surgery shows promising results in anterior and bi-leaflet MVP repair [39], Barlow’s disease, including multiple segmental prolapses and anterior leaflet involvement, may potentially be more difficult to repair [40] and is associated with a higher risk for redo surgery due to MR recurrence [41], particularly in the presence of a MAD [27].

(4) The patient preferred to avoid surgical intervention if possible.

**Figure 4:**
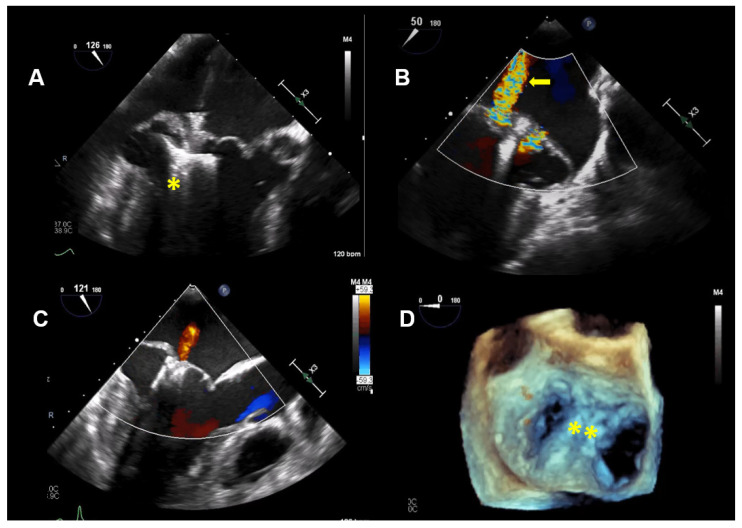
Case 2: Procedural transesophageal echocardiographic imaging during MitraClip XTR (Abbott Vascular, North Chicago, Illinois, USA) implantation. (**A**) The first MitraClip XTR (yellow asterisk) is positioned below the mitral leaflets with opened Clip arms. (**B**) Residual mitral regurgitation (MR) after implantation of the first Clip is marked with a yellow arrow. (**C**) After positioning of the second Clip, only mild MR is present. (**D**) The 2 MitraClip XTR devices (yellow asterisk), which create a double orifice, are seen in a 3D enfaced view.

**Figure 5:**
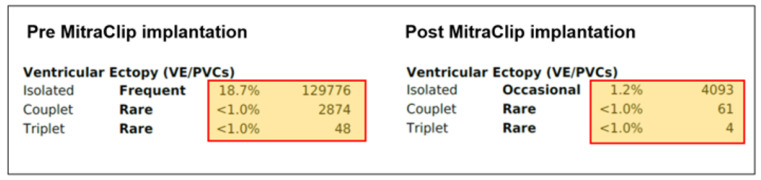
Case 2: Overview of the ventricular ectopies recorded by Zio XT patch monitoring (iRhythm Technologies, San Francisco, CA, USA) pre-MitraClip (**left**) and post-MitraClip XTR implantation at 1-year follow-up (**right**). PVCs: premature ventricular contractions.

Thus, based on all of the findings, an increased perioperative morbidity in this MR patient was hypothesized and, also, to comply with the patient not wanting surgery, the decision was made to proceed with a percutaneous MV repair strategy. Subsequently, two MitraClips (XTR) (Abbott Vascular, North Chicago, Illinois, USA) were implanted with an excellent result, with minimal residual MR (Figure 4). One year after MitraClip implantation, the patient reported significantly fewer palpitations, and her exercise tolerance was normal. TTE imaging documented normal LV function with a LVEF of 60%, normal pulmonary artery systolic pressure and mild, late-systolic MR. Using Zio XT patch monitoring, a marked reduction in PVCs from 18.7% to 1.2% and rare SVT were seen, and no nsVT occurred (data are in Figure 5).

## 3. Discussion

### 3.1. Case 1

In the first patient with a MAD and myxomatous MVP, the arrhythmogenic event was clinically in the foreground, while the MR was erroneously assessed as being severe. The echocardiographic evaluation of patients with MR by an MVP should carefully assess whether the MR is holosystolic or purely mid-late systolic. MR that occurs only mid-late systolic and not holosystolic is unlikely to be severe or to create hemodynamic consequences and is associated with a more benign course and more favorable long-term outcomes [42]. The evaluation of MR by means of the color flow area, vena contracta, and effective regurgitant orifice area (EROA) by flow convergence is problematic in this context. Although the MR jet size may appear similar, the regurgitation volume in mid-late systolic MR is significantly lower than in holosystolic MR [42]. It has also been shown that absolute EROA by flow convergence is not linked to the outcome in patients with mid-late systolic MR [42]. The regurgitant volume is more reflective of MR severity, in this case, and should be determined by multiplying the ERO with the MR velocity–time integral [42].

Van Wijngaarden et al. demonstrated that symptomatic ventricular arrhythmias are not uncommon in patients with an MVP (11% of patients) and are associated with significant annulus abnormalities, such as a MAD and annulus dilatation. However, severe arrhythmias (with an increased risk of SCD) were primarily found in a selected subgroup of patients—namely, young women with Barlow’s syndrome (generally without relevant MR)—while the overall risk was rather low in patients with an MVP (3%), suggesting that these patients should be consistently monitored diagnostically but the risk of SCD is low [43].

However, there is growing evidence that myxomatous MVP and MAD are associated with an increased risk of VTs and SCD [13,16], as seen in the patient described here.

Carmo et al. found that the severity of MAD was significantly correlated with the occurrence of nsVT on Holter monitoring. A disjunction distance >8.5 mm was thereby a strong predictor for nsVT [16].

In a further study involving 36 patients with arrhythmic MVP (with complex ventricular arrhythmias consisting of ventricular fibrillation and VT, either nonsustained or sustained) with concomitant LV fibrosis with LV late-gadolinium enhancement on CMR and no or trivial MR and 16 MVP patients without LV late-gadolinium enhancement examined with morpho-functional CMR, the MAD was identified as constant feature of arrhythmic MVP with LV fibrosis [13]. In patients with MVP and LV late-gadolinium enhancement, a significantly higher prevalence of mid-systolic click (72% versus 38%; *p* = 0.018), late-systolic murmur (69% versus 25%; *p* = 0.003), complex ventricular arrhythmias originating from the LV (89% versus 6%; *p* < 0.001), and the presence of bi-leaflet MVP (72% versus 31%; *p* = 0.005) was found as compared with those without late-gadolinium enhancement. Moreover, patients with MVP and late-gadolinium enhancement had a larger disjunction distance (median: 4.8 versus 1.8 mm; *p* < 0.001). A larger disjunction distance was also associated with a higher prevalence of SCD in the patient group with MVP and LV fibrosis [13].

Thus, it can be postulated that the excessive mobility of the mitral leaflets in the presence of MMVD and MVP in the area of the posterior leaflet, which is augmented by MAD, leads to increased mechanical stress of the posterolateral LV wall and the papillary muscles, which may lead to LV localized myocardial fibrosis, the morphological substrate of arrhythmic MVP [44,45].

A recently published study including 116 patients with MAD found that ventricular arrhythmias were frequent in patients with MAD. Of note, 22% of the patients with MAD did not have MVP, and MVP was not associated with the arrhythmic events. This is consistent with MAD being itself an arrhythmogenic entity [12].

Thus, a careful assessment of MV anomalies, including MAD in particular, especially in association with an auscultatory audible mid-systolic click, may help to better characterize patients who would benefit from risk stratification for significant arrhythmias. In this context, CMR helps to identify patients with myocardial fibrosis and, thus, an increased risk for arrhythmogenic events. It also means that MMVD, MVP, and MAD should be ruled out in patients with unexplained palpitations, especially younger ones.

### 3.2. Case 2

The second case focuses on the severity of MR and the need for treatment in a relatively young, symptomatic patient with advanced MMVD, bi-leaflet MVP, and MAD.

According to the current societal guidelines (ACC/AHA and European), chronic symptomatic severe MR is an indication for surgery, preferably MV repair [46,47], with good long-term results (at 20 years, reoperation-free survival was 60.4%) [48]. However, Newcomb et al. [27] reported that advanced myxomatous degeneration is an independent predictor of MV repair failure mainly due to an insufficient annuloplasty, to which a MAD can contribute significantly. In the presence of a MAD, the annuloplasty ring cannot adequately relieve the mechanical stress on the mitral leaflets, chordae, and papillary muscles. To make an annuloplasty effective, in this case, the posterior leaflet would have to be detached and reattached to the proximal musculature of the LV and then secured with an annuloplasty. Using such an approach, the probability of repair in patients with advanced MMVD and MAD was approximately 87%, as reported by Newcomb et al. [27]. However, the role of the displaced mitral annulus in patients with advanced MMVD on the long-term outcomes of MV repair is unknown. In addition, next to the complexity of MV morphology, other factors were present in this patient that could negatively influence the postoperative outcome, such as the presence of myocardial fibrosis [34,35,36] and the lack of a contractile reserve [37,38]. The increased perioperative risk in the overall context led to the decision to treat the patient with a percutaneous edge-to-edge approach by using XTR MitraClips. The NTR and XTR (Abbott Vascular, North Chicago, Illinois, USA) are two technically modified MitraClip versions that became available in 2018. Modifications were made to the delivery catheter by increasing the shaft extension by 1.5 cm, thus allowing for a more direct and better predictable trajectory during positioning and the lock mechanism. Compared to the NTR, the MitraClip XTR arm length has been increased from 9 mm to 12 mm, which improves coaptation and facilitates grasping by two additional rows of frictional elements at the grippers. Due to its technical features, and especially the longer Clip arms, the XTR is particularly suitable for extended prolapses with redundant leaflet tissue, and their successful use in advanced MMVD has already been reported [49]. The fourth generation of the MitraClip (G4), currently under limited release, includes two newer Clip sizes with 50% wider arms (referred to as NTW and XTW), which may be more beneficial in achieving a significant and durable reduction in MR patients with redundant leaflet tissue. The procedure regarding MR reduction was successful in this patient with persistently stable residual MR, as seen at the one-year follow-up.

As the degenerative process may persist even after MV repair, achieving MR reduction by edge-to-edge repair alone may not be sufficient in the long term in the presence of MAD, and further studies are needed to clarify whether additive annular therapy for MAD is necessary in these cases. For this reason, MAD should not be overlooked in patients with MMVD and MVP undergoing MitraClip implantation.

In addition to the MR reduction, there was a significant reduction of ventricular arrhythmias observed after MitraClip implantation, and LV function also improved. This may indicate that, with adequate reduction of MR, the mechanical stress on the mitral leaflets and, consequently, on the mitral annulus, the chordae, and the papillary muscles, diminishes even in the presence of MAD, thus minimizing, or at least reducing, a trigger for ventricular arrhythmias in the presence of a morphological substrate (the myocardial fibrosis).

Ledwoch et al. [50] recently demonstrated in a prospective study using MitraClips in patients with heart failure and severe MR (82% had secondary MR) a significant reduction in ventricular arrhythmias (nsVT and sustained ventricular tachycardia (sVT)). They were reduced from 32% to 14% at follow-up (*p* = 0.01), and the PVC decreased from 16% to 4% (*p* = 0.04). Patients with persistent or new nsVT and/or sVT at follow-up showed a significant decrease in LVEF from 38% to 33%. Whether these data are applicable to patients with MMVD and MAD is unclear. Studies are needed to better assess in MMVD with MVP and MAD to what extent ventricular arrhythmias can be reduced by sufficient MR reduction from surgery, as well as by MitraClip implantation. The impact on LV function also appears important.

## 4. Conclusions

The causal relationships between myxomatous MVP, MAD, and the mitral annulus are not yet fully understood. However, it is evident that the presence of a MAD in patients with MMVD has both functional and clinical implications and contributes to an increased incidence of ventricular arrhythmia and SCD. Thus, a MAD should not be overlooked in patients with MMVD, especially as this may influence the choice of therapy. The two cases we reported demonstrated the complexity of the decision-making process regarding therapy, especially when relevant MR is present, as these are mostly younger patients. The clinician should integrate the clinical, laboratory, and imaging findings. In this context, CMR provides important data on the severity of MR, effect on LV remodeling, and myocardial fibrosis. Surgical MV repair is standard for young patients with severe degenerative MR. However, transcatheter edge-to-edge repair can be a safe and effective option for high-risk patients. Whether the elimination of relevant MR (either surgically or by using a transcatheter approach) in the presence of a MAD is an effective long-term therapy needs to be confirmed, ideally in a prospective randomized controlled study.

## Figures and Tables

**Table 1:** Incidence of a MAD using different imaging modalities.

Reference	Number of Patients (*n*)	Imaging Modality	Length Used to Define MAD	Mad Incidence	MAD Length	Circumferential Extension of MAD	Additional Morphological Findings
Carmo et al. [16]	38 with MMVD	2D TTE	any length	55% of patients with MMVD had MAD	7.4 ± 8.7 mm	n.a.	Mitral annular function was significantly impaired in patients with MAD in whom the mitral annular diameter was paradoxically larger in systole than in diastole. The most severely affected patient had a MAD length of 30 mm and an involvement of the entire circumference.
Konda et al. [17]	185 (165 with FED, 10 with bi-leaflet prolapse and 10 with Barlow’s syndrome)	2D TTE	≥2 mm	24% of the patients with FED had MAD and 90% of the patients with MMVD.	MMVD: 4.34 ± 2.00 mm FED: 3.21 ± 0.74 mm	n.a.	The prolapse was located posteriorly in 77.8% of cases. Of the 51 patients with anterior MVP, only 1 had MAD. Among 111 patients with posterior MVP, 30.6% had MAD. None of the 3 patients with commissural site prolapse had MAD. Among 10 patients with Barlow’s syndrome, 90% had MAD.
Mantegazza et al. [25]	979 (342 with FED, 637 with MMVD)	2D TTE	any length	5.8% of patients with FED had MAD and 21.8% of patients with MMVD	FED: 5.9 ± 2.2 mm; MMVD: 6.7 ± 2.2 mm	n.a.	MAD was located in 69.2% in the lateral (P1) and middle (P2) segments of the posterior leaflet
Eriksson et al. [26]	67 with MMVD	2D TEE	≥5 mm	98% of patients with advanced MMVD had MAD whereas only 9% of patients with moderate MMVD had MAD	10 ± 3 mm	n.a.	A significant correlation between MAD distance and number of prolapsing segments was found.
Lee et al. [14]	101 with MVP	RT-3D TEE	≥5 mm	42% of patients with a MVP had MAD	8.9 mm (6.3–10.7 mm)	87° ± 41°	The 3D extent of MAD correlated significantly with abnormal annular dynamics and larger regurgitant orifice.
Putnam et al. [29]	90 with MVP	CCT	any length	20% of patients with a MVP had MAD	5.5 mm (3.8–9.6 mm)	n.a.	MAD was typically located adjacent to a prolapsed segment of the posterior leaflet.
Mantegazza et al. [30]	131 with MVP	CMR	any length	42% of patients with MVP had MAD	5 mm (4–7 mm)	n.a.	Good agreement on MAD measurements between TEE and CMR (moderate between TTE and CMR).
Dejgaard et al. [12]	116 with MAD	CMR	any length	MVP was evident in 78% of patients with MAD	3 mm (1–7 mm)	Median 150° (30°–240°)	MAD was exclusively found along the posterior leaflet.

TTE = transthoracic echocardiography, TEE = transesophageal echocardiography, 2D = two-dimensional, 3D = three-dimensional, RT = real-time, CCT = cardiac compute tomography, CMR = cardiac magnetic resonance, MAD = mitral annular disjunction, MMVD = myxomatous mitral valve disease, MVP = mitral valve prolapse, FED = fibroelastic deficiency, and n.a. = not available.

## Abbreviations

2D	two-dimensional
3D	three-dimensional
AICD	automatic implantable cardioverter defibrillator
CCT	cardiac computed tomography
CMR	cardiac magnetic resonance
ECG	electrocardiogram
EF	ejection fraction
EROA	effective regurgitant orifice area
LV	left ventricle
MAD	mitral annular disjunction
MMVD	myxomatous mitral valve disease
MR	mitral regurgitation
MV	mitral valve
MVP	mitral valve prolapse
nsVT	nonsustained ventricular tachycardia
sVT	sustained ventricular tachycardia
PVC	premature ventricular contractions
RT	real-time
SCD	sudden cardiac death
SVT	supraventricular tachycardia
TEE	transesophageal echocardiography
TTE	transthoracic echocardiography
VT	ventricular tachycardia
